# Broadband Collision-Induced Dissociation Mass Spectrometry Imaging

**DOI:** 10.1021/jasms.5c00045

**Published:** 2025-06-19

**Authors:** Sumi Krupa, Wiktoria Szuberla, Joanna Nizioł, Anna Ossolińska, Krzysztof Ossoliński, Tomasz Ruman

**Affiliations:** 1 Doctoral School at the Rzeszów University of Technology, 8 Powstańców Warszawy Ave., Rzeszów 35-959, Poland; 2 Department of Inorganic and Analytical Chemistry, Faculty of Chemistry, Rzeszów University of Technology, 6 Powstańców Warszawy Ave., Rzeszów 35-959, Poland; 3 Department of Polymers and Biopolymers, Faculty of Chemistry, 69696 Rzeszów University of Technology, 6 Powstańców Warszawy Ave., Rzeszów 35-959, Poland; 4 Department of Urology, John Paul II Hospital, Grunwaldzka 4 St., Kolbuszowa 36-100, Poland

**Keywords:** tandem mass spectrometry, mass spectrometry imaging, metabolomics, tissue imaging, molecular imaging

## Abstract

This study demonstrates the first application of broadband collision-induced dissociation (bbCID) to mass spectrometry imaging (MSI) for the identification of untargeted metabolites in human tissues. The methodology integrates bbCID with laser ablation–remote atmospheric pressure photoionization/chemical ionization (LARAPPI/CI), enabling the simultaneous acquisition of precursor and fragment ion distributions during MSI for many compounds simultaneously. In this approach, an infrared (IR) laser is used to ablate biological material, which is then ionized in the gas phase by a combination of photoionization and chemical ionization at atmospheric pressure. The method was validated using reference compounds, including thymidine and commonly used synthetic dyes, to assess ionization efficiency, fragmentation behavior, and spatial colocalization of precursor and fragment ions. Subsequently, bbCID-MSI was applied to clinical tissue samples of human bladder and kidney cancer. For the bladder cancer tissue, higher intensities of heptadecanoic acid, docosahexaenoic acid (FA(22:6)), docosapentaenoic acid (FA(22:5)), and FA(16:0) were observed in tumor regions, whereas proline was more abundant in adjacent nontumorous area. In renal cell carcinoma, cancerous regions exhibited elevated levels of polyunsaturated fatty acids such as arachidonic acid (FA(20:4)) and adrenic acid (FA(22:4)), while creatine and serine were enriched in healthy tissue zones. These findings highlight the utility of bbCID-MSI for spatially resolved metabolite analysis and its potential to reveal biologically relevant metabolic alterations associated with cancer.

## Graphical abstract

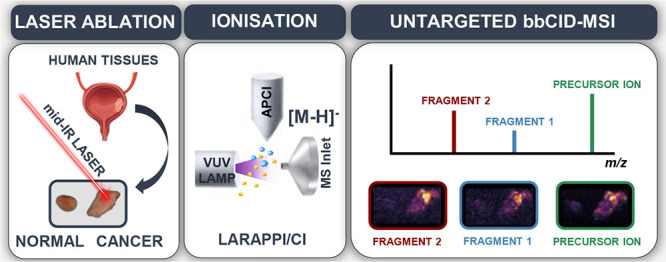

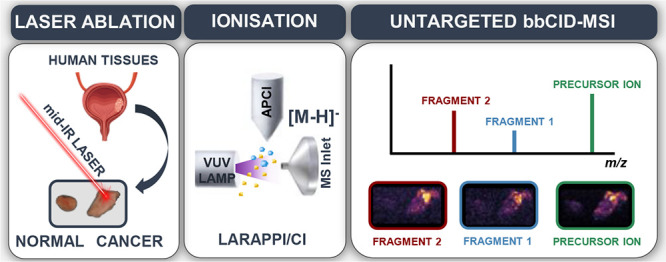

## Introduction

Mass spectrometry imaging (MSI) is a well-established analytical method that presents the spatial distribution of chemical compound ions that occur in biological tissues and synthetic materials. MSI has a plethora of applications in various fields, such as clinical, metabolic pathway research, and also biomarker discovery, microorganism activity, plant research, or forensic analysis.^1^

Most mass-spectrometry-based imaging analyses focus on acquiring results from the MS^1^ mass spectrum.^2^ The identification of compound ions detected by this method can be challenging for compounds of similar monoisotopic masses and very problematic for isomers. The solution to more accurate identification is the analysis of fragment ions in tandem mass spectrometry (MS/MS).^3^

The identification of detected ions with MSI and MS/MS is usually done by comparing the ion images obtained for precursors with the ion images recorded for fragment ions. The majority of published results present methodologies capable of the fragmentation of only one ion per analysis. These MS/MS analyses are focused on 3 main areas: lipid imaging in animal brain tissues,^4–6^ plant-specific metabolites,^7,8^ and drug imaging, including already existing drugs, a notable number conducted on brain tissues,^9–18^ drug candidates,^19,20^ and illicit drugs.^21–23^ The commercially available active drug substances studied using MSI range from antibacterial^24–26^ and antiviral drugs^12^ and allergy drugs^9,25,26^ to respiratory system drugs,^17,27,28^ anticancer drugs,^16,25,26^ and antipsychotic drugs,^10,13,15,25,26,29^ drugs on the skin,^30^ in rat brain tissue^31^ and rat lung tissue,^28^ and on disaccharide matrix.^32^ MS/MS imaging has also been utilized in forensic research for imaging fingerprints^33^ and forgery research.^34^

Selected reaction monitoring (SRM) and multiple reaction monitoring (MRM) are methods that allow mass spectrometry imaging by using multiple fragmentation reactions per analysis as a result of the relatively fast switching of *m*/*z* windows by quadrupole analyzers. However, the analysis with SRM/MRM MS or MSI is a targeted approach that requires knowledge of *m*/*z* values of precursors and fragment ions of compounds present in the tissue prior to analysis. SRM has been used in MSI analyses of tissues utilizing infrared laser ablation (LA) as the desorption method. SRM analyses of plant tissues, such as banana cross sections and spelled cross sections, have been conducted with MSI, allowing for the identification of amino acids, basic metabolites, plant-specific metabolites, and invading fungi metabolites.^35,36^ Objects such as 18th-century silk fabric^37^ and historic photographs^38^ have also been imaged by the discussed method, allowing the identification of microbial metabolites in negative and positive modes, respectively. Human kidney cancerous and healthy tissue has also been imaged in SRM mode with IR-LA desorption factor, leading to the identification of cancer-normal differentiating compounds.^39^

The use of a dedicated millijoule energy level IR laser as a desorption source is highly effective for biological samples and shows relatively deep penetration of biological material compared to other commonly used desorption methods, such as secondary-ion mass spectrometry (SIMS), matrix-assisted laser desorption/ionization (MALDI), and laser desorption/ionization (LDI) techniques, or desorption electrospray ionization (DESI).^39^ The main technique used for MS/MS mass spectrometry imaging reported at present, where infrared-LA is employed, is laser ablation–remote electrospray ionization (LARESI).^39^ LARESI is a technique advantageous for MSI due to numerous reasons such as (i) remote nitrogen-filled chamber with the sample, (ii) sample freezing, (iii) deep ablation, (iv) compatibility with all atmospheric ion sources and instruments, and (v) nonfragmenting low photon energy IR laser. Interferences to the composition and structures are minimal because of the minimal sample preparation and the ambient conditions of the analysis. While published studies employed triple quadrupole (QqQ) mass analyzers, which are widely recognized for their exceptional sensitivity and quantification capabilities, these instruments typically operate in a targeted mode and are not inherently designed for high-resolution data acquisition. The spectral resolution is a pivotal parameter in the accurate assignment of *m*/*z* value to a specific molecule.

Broadband collision-induced dissociation (bbCID) technology extends traditional CID workflows by employing a wide collision energy range and cycling from low to moderate fragmentation energy, thus generating simultaneous MS and MS/MS data for all suitable ions.^40^ Traditional CID typically targets only one selected precursor at a fixed collision energy, whereas bbCID fragments multiple ions and provides multilevel fragmentation, enabling rapid, nontargeted screening of complex mixtures.^41,42^ The bbCID as a data-independent approach (DIA) is usually used as a quantitative or qualitative method for metabolomic profiling of biological fluids and comprehensive screening of unknown contaminants.^43–45^ By capturing precursors and fragments in a single LC-HRMS run, bbCID allows detection and quantification of numerous analytes without repeated reinjection or sequential MS/MS acquisition steps.^46,47^

In the context of MSI, bbCID should provide several advantages. First, it provides both MS and MS/MS-level data simultaneously, bypassing the need for precursor selection. Second, its nontargeted fragmentation capability is particularly beneficial for detecting and identifying diverse classes of compounds for which fragmentation pathways may be unknown.^48–50^ Although wide-energy CID strategies have been successfully demonstrated in nonimaging MS for the characterization of complex biological samples,^51^ their adaptation to MSI platforms remains underexplored, highlighting the novelty of this work.

Laser ablation–remote atmospheric pressure photoionization/chemical ionization (LARAPPI/CI) technique provides several analytical advantages over widely adopted MSI platforms such as MALDI, DESI, and SIMS. The use of a mid-infrared (2.93 μm) pulsed laser enables efficient ablation of hydrated biological materials with minimal thermal degradation, allowing for depth profiling without the need for thin sectioning.^52^ Importantly, the system operates under ambient conditions and does not require matrix deposition, thereby reducing ion suppression and preserving the native distribution of analytes.^53^ Unlike MALDI, which relies on UV-absorbing matrices, and DESI, which requires solvent–surface interaction, LARAPPI/CI minimizes sample preparation while offering high chemical versatility and compatibility with both polar and nonpolar metabolites.^54^ Although SIMS achieves submicrometer spatial resolution, it is problematic for centimeter-sized samples and often suffers from low ion yields and limited performance in the high mass range for biomolecules. LARAPPI/CI delivers a practical balance between spatial resolution and ionization efficiency across diverse compound classes.^55^

In this study, we utilize a recently developed LARAPPI/CI system equipped with a high-mass-resolution quadrupole-time-of-flight (QToF) mass spectrometer. The mass spectrometry method employed is bbCID, which continuously cycles very low and high collision energies, enabling the acquisition of both MS and MS/MS-type data for potentially hundreds to thousands of organic compounds within a single MSI experiment. These capabilities are further enhanced by the integration of bbCID with LARAPPI/CI MSI, allowing the simultaneous acquisition of precursor and fragment ion distributions in each voxel, without the need for precursor selection. The resulting nontargeted, multienergy fragmentation profiles facilitate structural elucidation of unknown compounds directly within complex tissue environments, thereby supporting deeper metabolomic interpretation.

While bbCID has been extensively used in nonimaging applications, its adoption for MSI was not previously published. In the first part of this work, the methodology is validated using known reference compounds, while the second part provides results of application to the analysis of human normal and cancerous kidney and bladder tissues. The imaging method presented in this work was developed for Bruker’s bbCID but should also be compatible with other properly configured DIA methods, such as Thermo Fisher Scientific’s MS/AIF (full scan MS/all ion fragmentation) and Waters’ MS^E^.

## Materials and Methods

All solvents were LC-MS grade. All chemicals were purchased from Aldrich/Merck Poland and were of analytical purity.

### Patient Information

The kidney sample used in the MSI experiment was taken from a 72-year-old male. He was diagnosed with malignant clear cell renal cell carcinoma (ccRCC). The patient underwent a surgical procedure (NSS dex). The tumor size was 22 mm × 20 mm × 20 mm. The cancer stage was classified as pT1aN0M0, indicating an early stage without lymph nodes or distant metastases. The Fuhrman grade is G1, indicating a low level of cellular aggressiveness.^56^

The bladder cancer (BC) sample used in the MSI experiment originated from a 75-year-old male. The histopathological evaluation indicated the staging classification of pT0, suggesting an early stage of cancer of bladder tissue. The research was approved by the local Bioethics Committee at the University of Rzeszow (Poland, permit number 2018/04/10) and followed all current rules and regulations.

### LARAPPI/CI MSI System

The LARAPPI/CI MSI system first described in a recent publication is based on an airtight chamber pressurized with nitrogen gas to produce a nitrogen stream of 10 L/min. The sample is placed on a 50 mm × 50 mm sample stage, with a Peltier cooling plate that sustains the sample at −18 °C. The temperature-controlled sample stage is mounted on a motorized high-speed *XY*-stage The pulsed beam from the OPO laser (2.93 μm, 7 ns, 20 Hz, 2 mJ/pulse measured before the focusing lens) enters the sample chamber through the sapphire window, then the beam is expanded 3.75× and is redirected toward the sample stage by a gold mirror. The beam then goes through a diffractive optical element forming a square-shaped top-hat beam. It is then focused onto the sample surface by a 50 mm focal length aspherical ZnSe lens. The optical assembly and also the camera (FLIR Blackfly S, color camera, 6 MPix, Sony IMX178 sensor, lens, 12 mm C series fixed focal length lens, Edmund Optics, U.K.) with lens and distance sensor are mounted on aluminum rails and are in a fixed configuration; the only moving parts are *XYZ* stages. During imaging, the laser focal point remains fixed in space, whereas the sample is moved. A specially designed gas funnel is also a focusing assembly and is connected to a 6/4 mm (O.D/I.D.) PTFE tube. The overpressure in the chamber drives a 10 L/min nitrogen gas flow through the tube. The laser ablation plumes are entrained in the gas and transported to the modified ion source (Bruker VIP HESI in the APCI configuration) of the Bruker Impact II mass spectrometer. The ion source also had a VUV source (Hamamatsu L12542) mounted axially to the MS sampling cone inside the ion source. An HPLC pump (Agilent G1312A) provides a steady flow of a solvent mixture (1% toluene in methanol; 200 μL/min) to the APCI needle [1]. The settings of the ion source were as follows: APCI nebulizer, end plate offset 600 V, capillary 1000 V, corona 6000 nA, nebulizer 3.5 bar, dry gas 0.2 L/min, dry temperature 250 °C, probe gas temperature 350 °C, probe gas 4 L/min, exhaust turned on. Experiments were performed with the following settings: scan range *m*/*z* 47–1300, 4 Hz MS/bbCID frequency, and CID energy was cycled from 7 to 40 eV. ESI MS/MS spectra presented in Figures 1–3 and S1 are taken from spectral databases: NIST version 2023 ^57^ and HMDB 5.0.^58^ The details of software used for registration and visualization of results is described in our previous work.^55^

**Figure 1:**
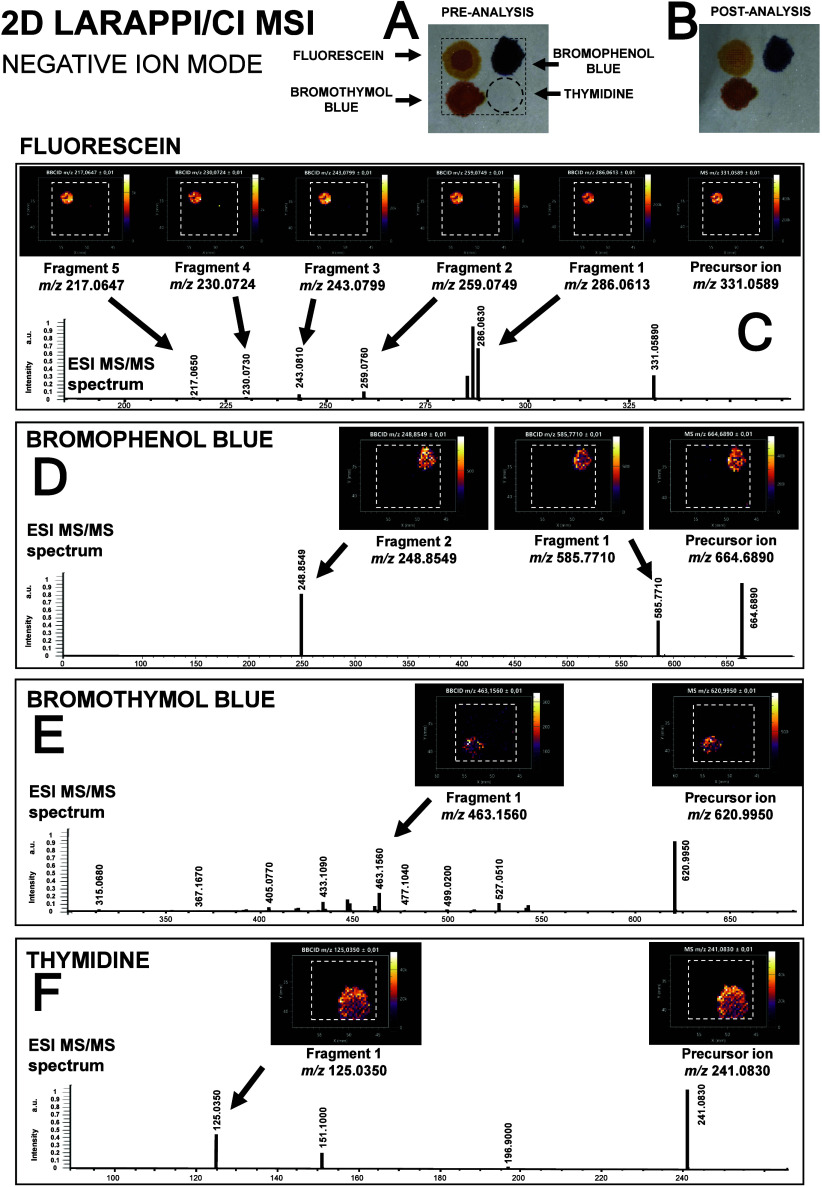
Results of negative ion mode LARAPPI/CI-MSI with bbCID method analysis of paper with fluorescein spots (upper left corner), bromophenol blue (upper right corner), bromothymol blue (lower left corner), and thymidine (lower right corner): (A) preanalysis optical photograph; (B) postanalysis optical photograph; (C–F) panels with ion images (top of the panel) and MS/MS spectra (bottom of the panel) for a given compound.

### MSI Experiment: Thymidine and Dyes on Paper

The analyzed compound spots were placed on laser printer paper (two layers, attached to a steel plate with adhesive tape) with automatic pipetting by pipetting 0.2 μL of each solution (thymidine 10 mg/mL in water, bromophenol blue, bromothymol blue, and fluorescein were of 50 mg/mL concentration in methanol).

The 2D MSI experiment in negative ion mode for dyes and thymidine was performed with 300 μm resolution (33 × 33 pixels, *X* × *Y*). Each pixel/voxel in 2D MSI experiments was exposed to the laser for 1 s at a laser pulse repetition rate of 20 Hz. The delays between pixels were 1000 ms. Between pixels, the sample stage moved at a speed of 50 mm/s. The time delay between the lines was 5 s. The starting object was of ca. 0.16 mm thickness and 9.6 mm × 9.6 mm (*X* × *Y*) size. The object was placed on a stainless steel plate, then placed on an ablation table inside the chamber, and frozen. The MS^1^ frequency was 3 Hz and the MS to bbCID spectrum recording time ratio was 1:1. The experiment in positive ion mode (Supporting Information Figure S1) was performed similarly to that described above with exceptions: number of pixels, 25 × 27; imaged region size, 7.2 mm × 7.8 mm (*X* × *Y*).

### MSI Experiment: Cancer and Normal Human Kidney Tissue

The 2D MSI experiment in negative ion mode for human kidney cancer and normal tissue was performed with a resolution of 150 μm (33 × 37 pixels, *X* × *Y*). Each pixel/voxel in the MSI experiment was exposed to the laser for 1 s at a laser pulse repetition rate of 20 Hz. The delays between pixels were 1000 ms. Between pixels, the sample stage moved at a speed of 50 mm/s. The time delay between lines was 5 s. The starting object was cut with a cryotome with *ca*. 0.5 mm thickness and the imaged area were 4.8 × 5.4 mm (*X* × *Y*) in size. The object was placed on a stainless steel plate and then on an ablation table inside the chamber and frozen. MS^1^/bbCID frequency was 4 Hz.

### MSI Experiment: Cancer and Normal Human Bladder Tissue

The 2D MSI experiment in negative ion mode for human bladder cancer and normal tissue was performed with 150 μm resolution (43 × 23 pixels, *X* × *Y*). Each pixel/voxel in the MSI experiment was exposed to the laser for 1 s, at a laser pulse repetition rate of 20 Hz. The delays between pixels were 1000 ms. Between pixels, the sample stage moved at a speed of 50 mm/s. The time delay between lines was 5 s. The starting object was cut with a cryotome with *ca*. 0.8 mm thickness and the imaged area were 6.3 mm × 3.3 mm (*X* × *Y*) in size. The object was placed on a stainless steel plate, then on an ablation table inside the chamber, and frozen.

## Results and Discussion

The use of classical fragmentation methods for untargeted MSI such as MS/MS where the precursor ion is selected manually or automatically is extremely impractical in fast imaging setups. In the case of our imaging setup LARAPPI/CI MSI, each ablation spot/voxel is usually ablated for just 0.3 to 1 s; this amount of time for MS/MS-type methods such as AutoMS/MS could allow signal detection, mass selection, and fragmentation of up to few ions with a fast modern instrument. However, the use of automatic MS/MS methods generates another critical problem, which is the differentiation of detection times for different voxels due to different amounts of signals being fragmented, which would artificially vary the MSI intensities of voxels and produce distorted results.

The method of choice for MSI was bbCID, which continuously cycles very low- and medium-high collision energy, allowing recording high-resolution spectra of both MS- and MS/MS type for large amounts of compounds in a single MSI analysis. The methodology in this work assumes improvement of MSI identification with matching of the ion image of precursor generated from MS data with the ion image(s) of fragments generated from bbCID data. Both data types are recorded during each MSI experiment. Four chemical compounds were chosen for preliminary tests: colorless thymidine, colorful bromophenol blue, bromothymol blue, and fluorescein. The choice of dyes allowed for a visual comparison of sample photographs with ion images. As the method was considered for research conducted on biological tissues, the fourth compound, thymidine, was chosen as an important biological compound to confirm the ionization and fragmentation of an endogenous metabolite. Dyes and thymidine samples were tested in 2D MSI in both negative (Figure 1) and positive (Figure S1) ion modes, with the former one chosen due to higher signal intensities. As the LARAPPI/CI MSI system contains a beam-shaping diffractive optical element, the focused laser spot size is a square with dimensions approximately 170 μm. The depth of the ablation of the paper object after 20 laser impulses was measured with a computerized microscope and was 60 μm. The calculated maximum weight of the desorbed material from a single ablation point for fluorescein was 30 ng, for bromophenol and bromothymol blue, 70 ng, and the mass of thymidine per singular spot was 1.5 ng, equating to no more than 1.2 nmol of bromothymol blue, 1.1 nmol of bromophenol blue, 1 nmol of fluorescein, and 60 pmol of thymidine.

All compounds were identified by their precursor ion [M – H]^−^, and at least one fragment signal was obtained for all compounds. The highest signals were obtained for fluorescein. The bbCID fragmentation of precursor ion of fluorescein of *m*/*z* 331.0589 produced 5 fragment ions with *m*/*z* values of 217.0647, 230.0724, 243.0799, 259.0749, and 286.0613 for which ion images are shown in Figure 1. For bromophenol blue, with the precursor ion at *m*/*z* 664.6890, two fragment ions were found: *m*/*z* 248.8549 and 585.7710. Both bromothymol blue and thymidine could be identified by one precursor ion and one fragment ion, *m*/*z* 620.9950/463.1560 and *m*/*z* 241.0830/125.0350 for precursor/fragment pairs of bromothymol blue and thymidine, respectively. The results obtained were deemed promising for further analysis of complex biological objects.

MSI is currently a key technique that enables the spatial analysis of metabolic changes within the tumor microenvironment.^59,60^ Its ability to perform untargeted molecular analysis without the need for labeling makes it particularly well-suited for cancer research, where metabolic reprogramming and tissue heterogeneity are frequently observed.^61^ In this study, we selected human bladder and kidney tissues containing both tumor and adjacent normal regions as a model system to evaluate the performance of the LARAPPI/CI-MSI-bbCID method under biologically complex conditions.

BC and normal bladder tissues were analyzed with the LARAPPI/CI-MSI-bbCID method (Figure 2). This type of comparison was chosen to differentiate cancer and normal regions based on possible cancer biomarkers. The compounds identified by comparison of MS and bbCID ion images were heptadecanoic acid (FA(17:0)), hexadecanoic acid (FA(16:0)), FA(22:6), FA(22:5) and proline, all of them can dissociate in water systems and form negative ions. For heptadecanoic acid, the *m*/*z* of the precursor and the most intense fragment was 269.2486/251.2385. Palmitic acid FA(16:0) anion at *m*/*z* 255.2320 could be identified by its fragment ions *m*/*z* 239.2011, 237.2218, and 225.1855. For proline *m*/*z* 114.0561 three fragment ions have been also detected: *m*/*z* 72.0086, 88.0399, 98.0242. Particularly good results were obtained for unsaturated fatty acids. Ion images of FA(22:6) precursor ion *m*/*z* 327.2329 matched ion fragment ion images of six fragments at *m*/*z* 107.0878, 121.1017, 135.1176, 175.1465, 183.1393, and 237.1861. FA(22:5) of the *m*/*z* 329.2486 of the precursor produced equally as many bbCID fragment ions at *m*/*z* 135.1174, 195.1385, 257.2269, 267.2113, 269.2269, and 285.2582 (Figure 2). It should be noted that all fragments discussed in this work are commonly found in CID MS/MS spectra (HMDB spectrum no. 21879, 32181, 21965, 2730, 3445, 2239403, 118936, 278884, 333573). Chemical structures of the precursors and fragments are provided in Figure 4. A study considering the fragmentation of saturated, monounsaturated, and polyunsaturated fatty acids has shown that saturated and monounsaturated FAs produce fragmentation spectra with significantly less distinguishable fragment signals than polyunsaturated acids,^62^ which may explain why some of the identified lipid species undergo more abundant fragmentation.

**Figure 2:**
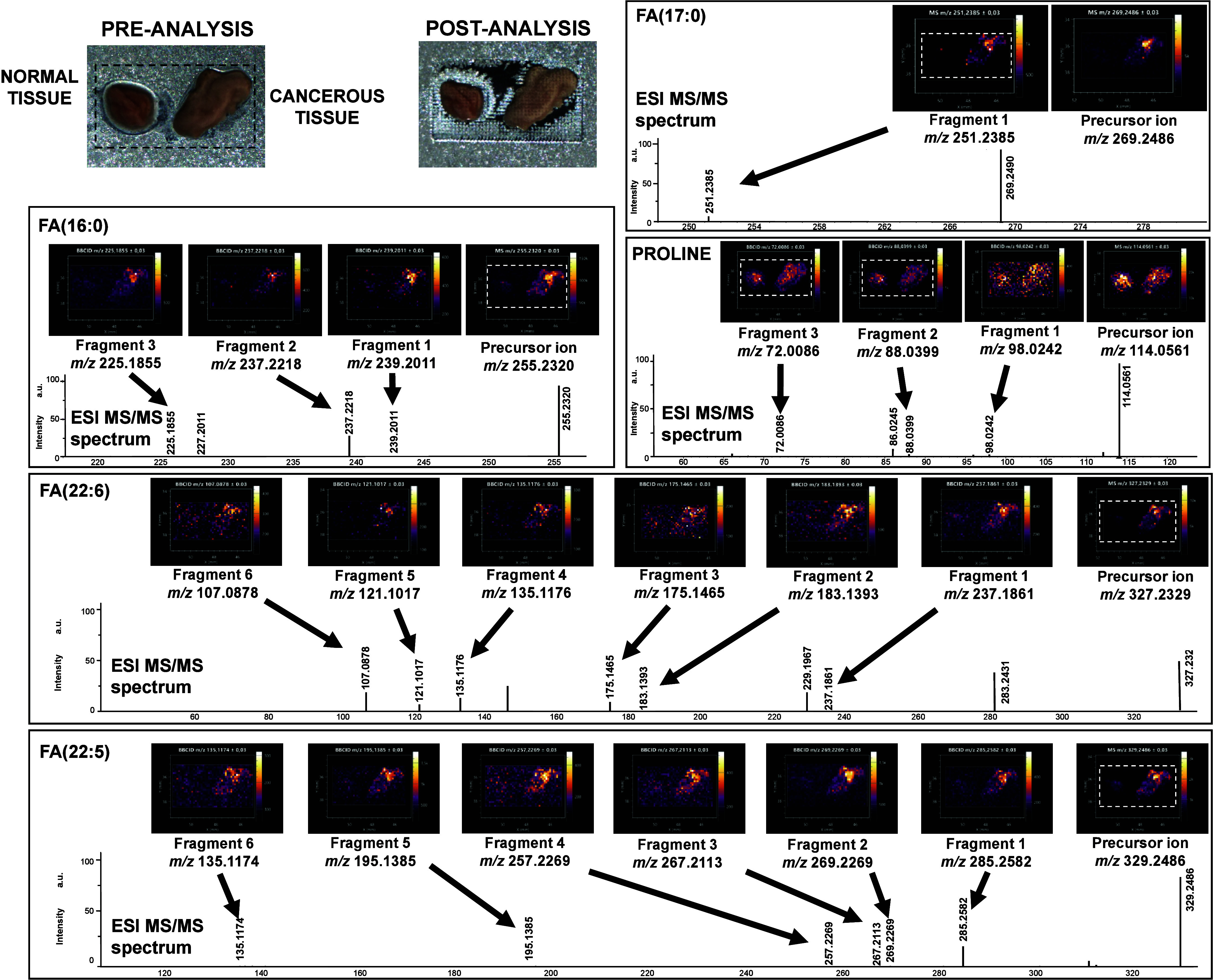
Results of LARAPPI/CI-MSI-bbCID analysis of BC tissue with the bbCID method. Preanalysis and postanalysis optical photographs of the studied object are shown in the top left corner of the figure. The rest of the figure contains panels with ion images (top of the panel) and MS/MS spectra (bottom of the panel) for the given compound. FA: fatty acid.

Heptadecanoic acid, although commonly present in human tissues, can be related to the amount of dairy intake in a diet,^63^ even though the ratio of pentadecanoic acid and heptadecanoic acid in dairy products versus in plasma of consumers of these products does not correlate in terms of concentration.^64^ Although pentadecanoic acid has a concentration about twice higher than that in dairy products, the opposite result is observed in human plasma after consumption. Heptadecanoic acid is sometimes attributed to a lower risk of coronary heart disease or type 2; however, studies show no significant differentiation in the obtained results.^65,66^ The ion images of heptadecanoic acid show the highest abundance of the compound in the cancerous tissue. Cancer research shows that heptadecanoic acid occurrence can be associated with a higher risk of prostate cancer,^67^ breast cancer,^68^ and colorectal cancer.^69^ Recent studies also suggest that heptadecanoic acid may induce apoptosis and enhance chemosensitivity in pancreatic cancer cells, further supporting its potential role in tumor pathophysiology.^70^

Palmitic acid (FA(16:0)) is one of the most abundant saturated fatty acids in human tissues, playing a central role in membrane integrity and energy metabolism.^71^ Recent studies have shown that elevated levels of palmitic acid can influence various oncogenic processes, including the induction of pro-inflammatory signals, modulation of oxidative stress, and promotion of tumor cell proliferation.^72,73^ Several studies have reported elevated levels of palmitic acid (FA(16:0) in cancer patients. In esophageal cancer, patients showed significantly higher plasma levels of FA(16:0) compared to healthy controls, indicating a potential role in tumor-related metabolic changes.^74^ Similarly, in colorectal cancer, both cachectic and noncachectic patients had increased plasma FA(16:0) levels compared to controls, suggesting its involvement in inflammation-associated cancer progression.^75^ A recent meta-analysis found that elevated blood levels of palmitic acid (FA(16:0)) were significantly associated with an increased risk of several cancers, including breast cancer.^76^ In bladder cancer, metabolic profiling has revealed elevated levels of palmitic acid (FA(16:0)) in tumor tissues, particularly in the basal subtype of muscle-invasive cases,^77,78^ suggesting its involvement in disease progression. Proposed mechanisms include activation of transcription factors like SREBP1, stimulation of pro-inflammatory pathways such as TLR-4/NF-κB, and epigenetic alterations affecting lipid metabolism, all of which may contribute to a tumor-promoting environment.^79^

The ion images of the proline ion show that the abundance of the compound is similar in both normal and cancerous tissue, with slightly higher levels in normal tissue. Previous metabolomic studies also suggest lower urinary proline concentrations in bladder cancer patients compared to healthy individuals.^80^ One possible explanation may involve disrupted proline biosynthesis, which occurs via the P5C reductase pathway and involves three isoforms: PYCR1, PYCR2, and PYCRL. Among these, PYCR1 has been implicated in increasing intracellular proline concentrations and supporting tumor growth in renal cell carcinoma.^81^ Beyond biosynthesis, recent studies demonstrate that proline catabolism is actively reprogrammed in cancer cells. Specifically, degradation of proline by proline dehydrogenase (PRODH) has been shown to facilitate metastasis formation in breast cancer models, while pharmacological inhibition of PRODH suppresses lung metastases without significantly impacting primary tumor size.^82^ Moreover, recent metabolomic studies of bladder cancer further support the relevance of proline dysregulation. For instance, altered proline levels were identified among metabolites distinguishing low- from high-grade bladder tumors in serum^83^ and urinary proline was among key discriminators in a diagnostic panel with high predictive accuracy.^84^ These findings highlight the dual regulatory roles of proline turnover (both synthetic and catabolic) in promoting tumor progression in a context-dependent manner.

Polyunsaturated fatty acids (PUFAs), including docosahexaenoic acid (FA(22:6)) and its metabolic precursor FA(22:5), were elevated in bladder cancer tissues compared to those in adjacent normal regions. FA(22:6) plays a multifaceted role in the tumor microenvironment due to its incorporation into membrane phospholipids, which not only enhances fluidity but also increases vulnerability to oxidative stress. This susceptibility contributes to its involvement in ferroptosis, a regulated form of cell death driven by reactive oxygen species (ROS) and iron.^85,86^ While elevated FA(22:6) can promote pro-ferroptotic activity and inhibit metastasis in certain cancers tumor cells with strong antioxidant defenses may exploit it to support survival.^87,88^ FA(22:5), although less studied, has been linked to immunomodulation and membrane remodeling. These findings align with recent reports on the role of PUFAs in metabolic reprogramming and redox regulation in cancer.^89^ Additionally, FA(22:6) has been shown to suppress bladder cancer cell invasiveness by downregulating granzyme B expression,^90^ while reduced expression of elongation of very long chain fatty acids protein 2 (ELOVL2), a key enzyme involved in DHA synthesis, has been associated with advanced-stage tumors and poor prognosis.^91^

The second human tissue analyzed in this study was the RCC and adjacent normal kidney tissue (Figure 3). Chemical structures of detected precursors and fragments are listed in Figure 4. Compounds identified with comparison of precursor ion images from MS spectra and fragment ions from bbCID spectra were creatine, FA(20:4), FA(22:4), and serine. Creatine and serine were identified by one precursor ion and one fragment ion, *m*/*z* 130.0625/88.0406 and *m*/*z* 104.0353/74.0250, respectively. Once again, the best results were obtained for polyunsaturated fatty acids. FA(20:4) *m*/*z* 303.2329 was identified by well-fitting ion images of the precursor ion and fragment ions *m*/*z* 177.1651 and 205.1963. For FA(22:4) *m*/*z* 331.2490, the assigned fragments found were *m*/*z* 259.2456 and 329.2494.

**Figure 3:**
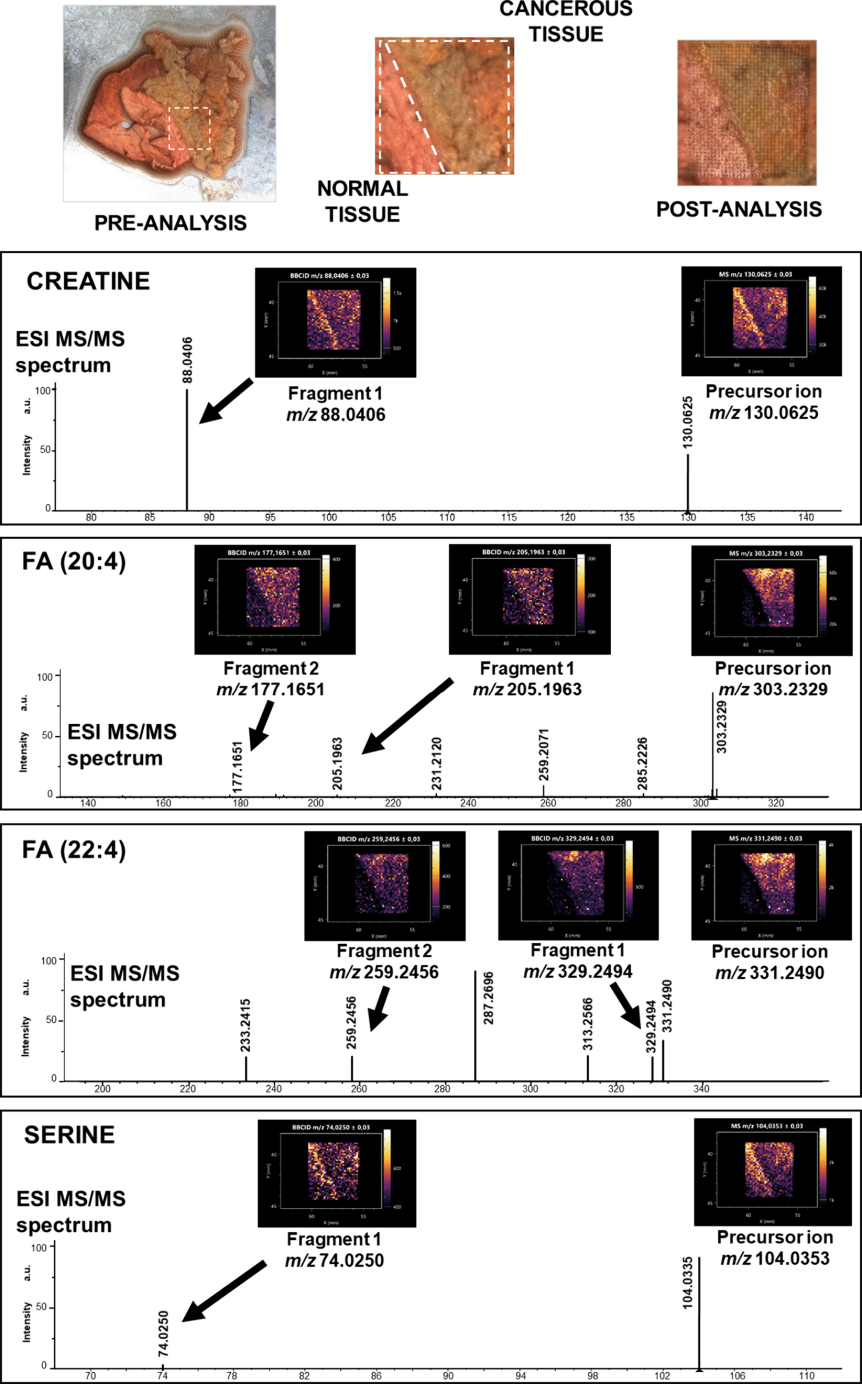
Results of LARAPPI/CI-MSI-bbCID analysis of human RCC tissue. Preanalysis optical photographs of the studied object are shown in the top-left and top-center parts of the figure. The postanalysis optical photograph is shown in the top right corner of the figure. The rest of the figure contains panels with ion images (top of the panel) and MS/MS spectra (bottom of the panel) for the given compound. FA: fatty acid.

**Figure 4:**
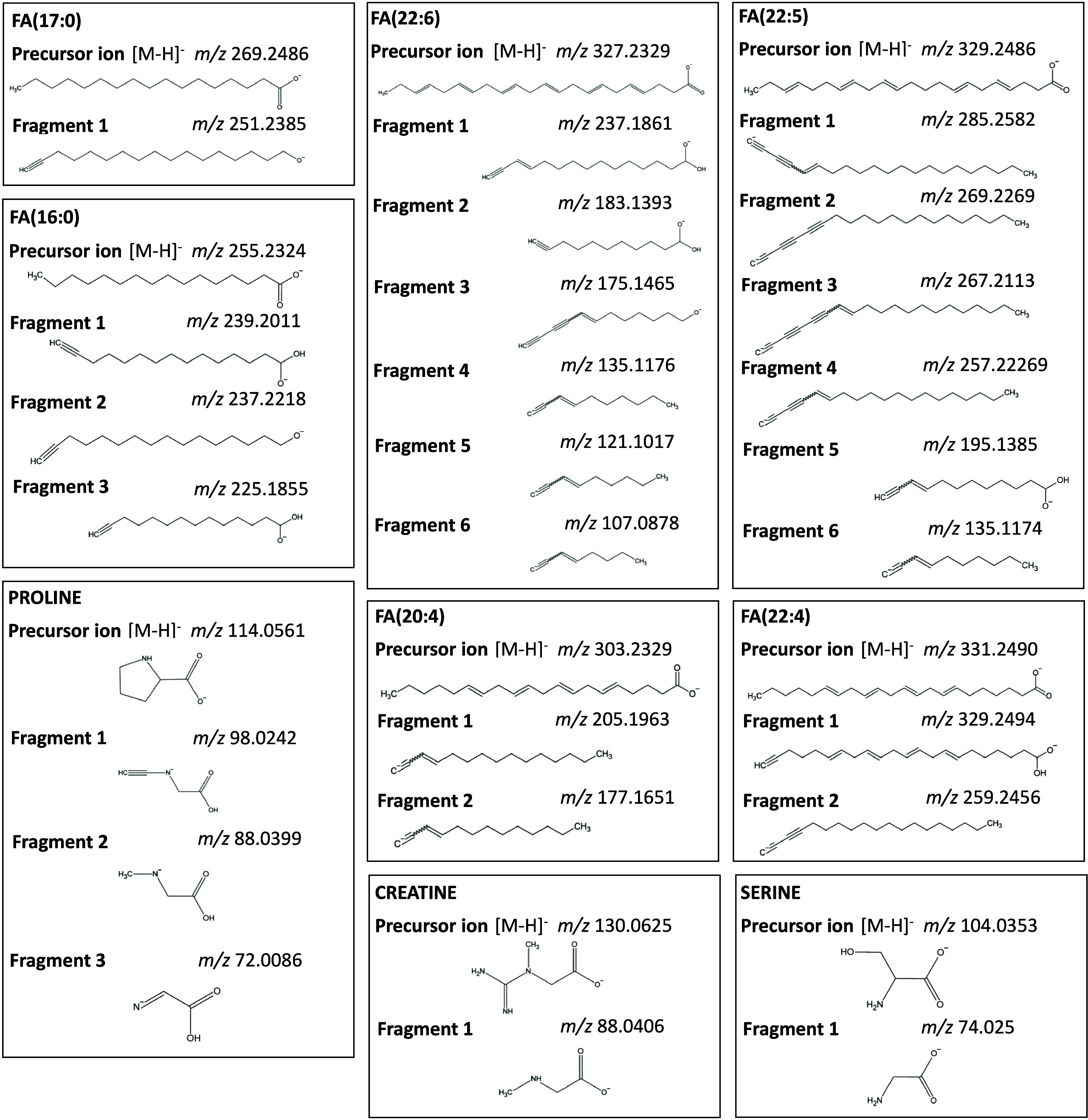
Chemical structures of ions detected in MSI experiments of the RCC and BC tissues. Each panel contains structure and *m*/*z* for precursor ion found in MS spectra (top of panel) and the same data for fragment ions found in bbCID spectra (middle and lower parts of panel). FA: fatty acid.

In our study, creatine showed the highest levels in the pseudocapsule, the zone between cancerous and healthy kidney tissue. Creatine is important for energy metabolism, and the enzyme creatine kinase helps convert it to phosphocreatine, which stores energy in cells. This enzyme has been linked to cancer progression in several tumor types, including gastric, esophageal, lung, and breast cancer.^92^ Higher levels of phosphocreatine can help cancer cells survive in stressful conditions by quickly supplying energy when needed.^93,94^ Metabolomic profiling of kidney cancer tissues has shown that creatine levels tend to be lower in malignant regions compared to adjacent noncancerous tissue,^95^ supporting the notion that creatine is more abundant in metabolically normal areas. This distribution pattern may result from differences in creatine transport, utilization, or local enzymatic activity. These findings are consistent with studies in other cancers, where MSI has shown an uneven creatine distribution between tumors and surrounding tissues. In breast cancer, energy-buffering pathways involving creatine and related metabolites have been linked to tumor adaptation under metabolic stress.^96^ Similarly, brain MSI data indicate that creatine levels shift in response to physiologic challenges, highlighting its role in energy homeostasis.^97^

Serine was also more abundant in noncancerous kidney regions, consistent with reports of altered serine biosynthesis in RCC due to upregulation of enzymes like phosphoglycerate dehydrogenase (PHGDH) and serine hydroxymethyltransferase 2 (SHMT2), which participate in serine–glycine one-carbon metabolism pathways critical for redox balance and biosynthesis.^98^ Lower levels of serine in malignant regions were confirmed in two independent RCC tissue samples that contained both cancerous and adjacent normal areas, using two MSI techniques, LARESI-SRM-MSI and ^109^AgNPET-LDI-MSI.^39^ In both cases, serine signals were consistently stronger in the noncancerous regions. This observation supports the notion that serine depletion in tumors may reflect increased utilization for anabolic growth and folate cycle activity. Furthermore, studies have linked elevated expression of serine biosynthetic enzymes with poor prognosis and therapeutic resistance in multiple cancer types, highlighting serine metabolism as a potential therapeutic target. However, previous studies have also highlighted the importance of serine metabolism in cancer, with increased serine levels reported in bladder cancer tissue^99^ and leukemia cell lines^100^ and decreased levels observed in urine samples of RCC patients,^56^ underscoring the importance of sample type when interpreting metabolite distribution.

The renal cancer samples showed increased abundance of arachidonic acid (FA(20:4)) and adrenic acid (FA(22:4)), both ω-6 PUFAs involved in inflammation and tumor progression.^101,102^ FA(20:4) serves as a precursor for a variety of eicosanoids, bioactive lipids that modulate angiogenesis, immune responses, and cell proliferation.^103^ Its elevated levels have been associated with increased tumor growth and poor prognosis in various malignancies.^104^ FA(22:4), synthesized from FA(20:4) via elongation, may amplify these effects by serving as an alternative substrate for lipid signaling or by promoting membrane lipid peroxidation.^105^ Notably, both FA(20:4) and FA(22:4) have been identified as potential modulators of ferroptosis sensitivity depending on the cellular oxidative environment, supporting their relevance in tumor pathophysiology.^105^ Our findings align with previous MSI-based analyses of RCC tissue, which also demonstrated elevated arachidonic acid levels in tumor regions compared to adjacent normal kidney.^106^ Increased FA(20:4) signals were localized specifically within malignant zones, reinforcing its relevance as a spatially resolved metabolic marker of the RCC.

As results of tissue imaging originate from single patients in both RCC and BC cases, the observed metabolic differences should be interpreted with caution as they may not fully capture the biological variability present in larger patient cohorts. Nevertheless, the compounds highlighted in our analysis were systematically cross-validated against the existing literature data. All of the presented metabolites have previously been detected in human biological matrices and reported in association with cancer-related metabolic alterations. Furthermore, our findings are consistent with those of other studies that identified the same compounds in tissues from corresponding tumor types, lending additional support to the biological relevance of our results.

## Conclusion

A novel approach for untargeted mass spectrometry imaging with improved identification is presented. This method uses an IR laser for deep desorption of the material and atmospheric pressure chemical ionization and photoionization (LARAPPI/CI) for the ionization of ablated material in a gas phase. By simultaneously collecting full-scan (MS) and fragmentation (MS/MS) data for multiple analytes, bbCID maximizes information density in a single MSI experiment and substantially reduces the overall acquisition time, which is crucial for capturing metabolic heterogeneity and potential biomarker candidates in spatially complex tissues. For the first time, bbCID was shown as an MSI-compatible measurement method, allowing untargeted identification of endogenous compounds. The results of the measurements suggested that FA(17:0), FA(16:0), FA(22:6), and FA(22:5) are in higher levels in cancerous bladder cancer tissue, while proline levels are slightly higher in normal tissue. Compounds such as FA(20:4) and FA(22:4) are at higher levels in RCC tissues, and on the contrary, creatine and serine are at lower levels compared to normal kidney tissue.

## Supplementary Material



## Data Availability

The data sets generated during and/or analyzed during the current study are available from the corresponding author upon request and in the RepOD open data repository (DOI: https://doi.org/10.18150/XSUCCF).

## Notes

Results of positive mode LARAPPI/CI-MSI analysis, with ion images of precursor and fragment ions of each compound shown above the ESI MS/MS spectra (PDF)

## References

[1] Perez C. J., Bagga A. K., Prova S. S., Yousefi Taemeh M., Ifa D. R. (2019). Review and Perspectives on the Applications of Mass
Spectrometry Imaging under Ambient Conditions. Rapid Commun. Mass Spectrom..

[2] Sans M., Feider C. L., Eberlin L. S. (2018). Advances
in Mass Spectrometry Imaging
Coupled to Ion Mobility Spectrometry for Enhanced Imaging of Biological
Tissues. Curr. Opin Chem. Biol..

[3] Baquer G., Sementé L., Mahamdi T., Correig X., Ràfols P., García-Altares M. (2023). What Are We Imaging? Software Tools
and Experimental Strategies for Annotation and Identification of Small
Molecules in Mass Spectrometry Imaging. Mass
Spectrom Rev..

[4] Müller M. A., Kompauer M., Strupat K., Heiles S., Spengler B. (2021). Implementation
of a High-Repetition-Rate Laser in an AP-SMALDI MSI System for Enhanced
Measurement Performance. J. Am. Soc. Mass Spectrom..

[5] Prentice B. M., McMillen J. C., Caprioli R. M. (2019). Multiple TOF/TOF Events in a Single
Laser Shot for Multiplexed Lipid Identifications in MALDI Imaging
Mass Spectrometry. Int. J. Mass Spectrom..

[6] Fisher G. L., Bruinen A. L., Ogrinc
Potočnik N., Hammond J. S., Bryan S. R., Larson P. E., Heeren R. M. A. (2016). A New Method
and Mass Spectrometer Design for TOF-SIMS Parallel Imaging MS/MS. Anal. Chem..

[7] Angel P. M., Caprioli R. M. (2013). Matrix-Assisted
Laser Desorption Ionization Imaging
Mass Spectrometry: In Situ Molecular Mapping. Biochemistry.

[8] Korte A. R., Yandeau-Nelson M. D., Nikolau B. J., Lee Y. J. (2015). Subcellular-Level
Resolution MALDI-MS Imaging of Maize Leaf Metabolites by MALDI-Linear
Ion Trap-Orbitrap Mass Spectrometer. Anal Bioanal
Chem..

[9] Li F., Hsieh Y., Kang L., Sondey C., Lachowicz J., Korfmacher W. A. (2009). MALDI-Tandem Mass Spectrometry Imaging of Astemizole
and Its Primary Metabolite in Rat Brain Sections. Bioanalysis.

[10] Khatib-Shahidi S., Andersson M., Herman J. L., Gillespie T. A., Caprioli R. M. (2006). Direct Molecular Analysis of Whole-Body Animal Tissue
Sections by Imaging MALDI Mass Spectrometry. Anal. Chem..

[11] Prentice B. M., Chumbley C. W., Caprioli R. M. (2015). High-Speed MALDI MS/MS Imaging Mass
Spectrometry Using Continuous Raster Sampling. Journal of Mass Spectrometry.

[12] Barry J. A., Robichaud G., Bokhart M. T., Thompson C., Sykes C., Kashuba A. D. M., Muddiman D. C. (2014). Mapping Antiretroviral Drugs in Tissue
by IR-MALDESI MSI Coupled to the Q Exactive and Comparison with LC-MS/MS
SRM Assay. J. Am. Soc. Mass Spectrom..

[13] Wiseman J. M., Ifa D. R., Zhu Y., Kissinger C. B., Manicke N. E., Kissinger P. T., Cooks R. G. (2008). Desorption Electrospray
Ionization Mass Spectrometry: Imaging Drugs and Metabolites in Tissues. Proc. Natl. Acad. Sci. U. S. A..

[14] Jacobsen S. C., Speth N. R., Xiong M., Herth M. M., Kristensen J. L., Palner M., Janfelt C. (2021). Desorption
Electrospray Ionization
Mass Spectrometry Imaging of Cimbi-36, a 5-HT2A Receptor Agonist,
with Direct Comparison to Autoradiography and Positron Emission Tomography. Mol. Imaging Biol..

[15] Koeniger S. L., Talaty N., Luo Y., Ready D., Voorbach M., Seifert T., Cepa S., Fagerland J. A., Bouska J., Buck W., Johnson R. W., Spanton S. (2011). A Quantitation
Method for Mass Spectrometry Imaging. Rapid
Commun. Mass Spectrom..

[16] Marko-Varga G., Fehniger T. E., Rezeli M., Döme B., Laurell T., Végvári Á. (2011). Drug
Localization
in Different Lung Cancer Phenotypes by MALDI Mass Spectrometry Imaging. J. Proteomics.

[17] Fehniger T. E., Végvári Á., Rezeli M., Prikk K., Ross P., Dahlbäck M., Edula G., Sepper R., Marko-Varga G. (2011). Direct Demonstration
of Tissue Uptake of an Inhaled
Drug: Proof-of-Principle Study Using Matrix-Assisted Laser Desorption
Ionization Mass Spectrometry Imaging. Anal.
Chem..

[18] Manier M. L., Reyzer M. L., Goh A., Dartois V., Via L. E., Barry C. E., Caprioli R. M. (2011). Reagent Precoated Targets for Rapid
In-Tissue Derivatization of the Anti-Tuberculosis Drug Isoniazid Followed
by MALDI Imaging Mass Spectrometry. J. Am. Soc.
Mass Spectrom..

[19] Goodwin R. J. A., MacKay C. L., Nilsson A., Harrison D. J., Farde L., Andren P. E., Iverson S. L. (2011). Qualitative
and Quantitative MALDI
Imaging of the Positron Emission Tomography Ligands Raclopride (a
D2 Dopamine Antagonist) and SCH 23390 (a D1 Dopamine Antagonist) in
Rat Brain Tissue Sections Using a Solvent-Free Dry Matrix Application
Method. Anal. Chem..

[20] Reyzer M. L., Hsieh Y., Ng K., Korfmacher W. A., Caprioli R. M. (2003). Direct Analysis of Drug Candidates
in Tissue by Matrix-Assisted
Laser Desorption/Ionization Mass Spectrometry. Journal of Mass Spectrometry.

[21] Beasley E., Francese S., Bassindale T. (2016). Detection
and Mapping of Cannabinoids
in Single Hair Samples through Rapid Derivatization and Matrix-Assisted
Laser Desorption Ionization Mass Spectrometry. Anal. Chem..

[22] Pirman D. A., Reich R. F., Kiss A., Heeren R. M. A., Yost R. A. (2013). Quantitative
MALDI Tandem Mass Spectrometric Imaging of Cocaine from Brain Tissue
with a Deuterated Internal Standard. Anal. Chem..

[23] Porta T., Grivet C., Kraemer T., Varesio E., Hopfgartner G. (2011). Single Hair
Cocaine Consumption Monitoring by Mass Spectrometric Imaging. Anal. Chem..

[24] Boudon S. M., Morandi G., Prideaux B., Staab D., Junker U., Odermatt A., Stoeckli M., Bauer D. (2014). Evaluation of Sparfloxacin
Distribution by Mass Spectrometry Imaging in a Phototoxicity Model. J. Am. Soc. Mass Spectrom..

[25] Swales J. G., Strittmatter N., Tucker J. W., Clench M. R., Webborn P. J. H., Goodwin R. J. A. (2016). Spatial
Quantitation of Drugs in
Tissues Using Liquid Extraction Surface Analysis Mass Spectrometry
Imaging. Scientific Reports 2016 6:1.

[26] Swales J.
G., Tucker J. W., Strittmatter N., Nilsson A., Cobice D., Clench M. R., Mackay C. L., Andren P. E., Takáts Z., Webborn P. J. H., Goodwin R. J. A. (2014). Mass Spectrometry Imaging of Cassette-Dosed
Drugs for Higher Throughput Pharmacokinetic and Biodistribution Analysis. Anal. Chem..

[27] Nilsson A., Fehniger T. E., Gustavsson L., Andersson M., Kenne K., Marko-Varga G., Andrén P. E. (2010). Fine Mapping
the Spatial Distribution and Concentration of Unlabeled Drugs within
Tissue Micro-Compartments Using Imaging Mass Spectrometry. PLoS One.

[28] Végvári Á., Fehniger T. E., Gustavsson L., Nilsson A., Andrén P. E., Kenne K., Nilsson J., Laurell T., Marko-Varga G. (2010). Essential
Tactics of Tissue Preparation and Matrix Nano-Spotting for Successful
Compound Imaging Mass Spectrometry. J. Proteomics.

[29] Hsieh Y., Casale R., Fukuda E., Chen J., Knemeyer I., Wingate J., Morrison R., Korfmacher W. (2006). Matrix-Assisted
Laser Desorption/Ionization Imaging Mass Spectrometry for Direct Measurement
of Clozapine in Rat Brain Tissue. Rapid Commun.
Mass Spectrom..

[30] Marshall P., Toteu-Djomte V., Bareille P., Perry H., Brown G., Baumert M., Biggadike K. (2010). Correlation of Skin Blanching and
Percutaneous Absorption for Glucocorticoid Receptor Agonists by Matrix-Assisted
Laser Desorption Ionization Mass Spectrometry Imaging and Liquid Extraction
Surface Analysis with Nanoelectrospray Ionization Mass Spectrometry. Anal. Chem..

[31] Goodwin R. J. A., Scullion P., MacIntyre L., Watson D. G., Pitt A. R. (2010). Use of
a Solvent-Free Dry Matrix Coating for Quantitative Matrix-Assisted
Laser Desorption Ionization Imaging of 4-Bromophenyl-1,4-Diazabicyclo(3.2.2)
Nonane-4-Carboxylate in Rat Brain and Quantitative Analysis of the
Drug from Laser Microdissected Tissue Regions. Anal. Chem..

[32] Zhan L., Huang X., Xue J., Liu H., Xiong C., Wang J., Nie Z. (2021). MALDI-TOF/TOF Tandem Mass Spectrometry
Imaging Reveals Non-Uniform Distribution of Disaccharide Isomers in
Plant Tissues. Food Chem..

[33] Yagnik G. B., Korte A. R., Lee Y. J. (2013). Multiplex
Mass Spectrometry Imaging
for Latent Fingerprints. Journal of Mass Spectrometry.

[34] Hamid T. S., Lostun D., Cabral E. C., Garrett R., Bohme D. K., Ifa D. R. (2015). Comparisons of Ambient
Spray Ionization Imaging Methods. Int. J. Mass
Spectrom..

[35] Nizioł J., Misiorek M., Krupa Z., Ruman T. (2024). Infrared Laser-Based
Selected Reaction Monitoring Mass Spectrometry Imaging of Banana (Musa
Spp.) Tissue—New Method for Detection and Spatial Localization
of Metabolites in Food. Food Anal Methods.

[36] Szulc J., Ruman T. (2020). Laser Ablation Remote-Electrospray Ionisation Mass Spectrometry (LARESI
MSI) Imaging—New Method for Detection and Spatial Localization
of Metabolites and Mycotoxins Produced by Moulds. Toxins.

[37] Szulc J., Karbowska-Berent J., Dra̧żkowska A., Ruman T., Beech I., Sunner J. A., Gutarowska B. (2021). Metabolomics
and Metagenomics Analysis of 18th Century Archaeological Silk. Int. Biodeterior Biodegradation.

[38] Szulc J., Ruman T., Karbowska-Berent J., Kozielec T., Gutarowska B. (2020). Analyses of
Microorganisms and Metabolites Diversity on Historic Photographs Using
Innovative Methods. J. Cult Herit.

[39] Nizioł J., Sunner J., Beech I., Ossoliński K., Ossolińska A., Ossoliński T., Płaza A., Ruman T. (2020). Localization of Metabolites of Human
Kidney Tissue with Infrared
Laser-Based Selected Reaction Monitoring Mass Spectrometry Imaging
and Silver-109 Nanoparticle-Based Surface Assisted Laser Desorption/Ionization
Mass Spectrometry Imaging. Anal. Chem..

[40] Reveglia P., Agudo-Jurado F. J., Barilli E., Masi M., Evidente A., Rubiales D. (2023). Uncovering Phytotoxic Compounds Produced by Colletotrichum
Spp. Involved in Legume Diseases Using an OSMAC-Metabolomics Approach. J. Fungi.

[41] Edelson-Averbukh M., Pipkorn R., Lehmann W. D. (2007). Analysis
of Protein Phosphorylation
in the Regions of Consecutive Serine/Threonine Residues by Negative
Ion Electrospray Collision-Induced Dissociation. Approach to Pinpointing
of Phosphorylation Sites. Anal. Chem..

[42] White M. E.
H., Sinn L. R., Jones D. M., de Folter J., Aulakh S. K., Wang Z., Flynn H. R., Krüger L., Tober-Lau P., Demichev V., Kurth F., Mülleder M., Blanchard V., Messner C. B., Ralser M. (2024). Oxonium Ion Scanning
Mass Spectrometry for Large-Scale Plasma Glycoproteomics. Nature Biomedical Engineering 2023 8:3.

[43] Chen Y. C., Wu H. Y., Chang C. W., Liao P. C. (2022). Post-Deconvolution
MS/MS Spectra Extraction with Data-Independent Acquisition for Comprehensive
Profiling of Urinary Glucuronide-Conjugated Metabolome. Anal. Chem..

[44] Chang J. K., Teo G., Pewzner-Jung Y., Cuthbertson D. J., Futerman A. H., Wenk M. R., Choi H., Torta F. (2023). Q-RAI Data-Independent Acquisition
for Lipidomic Quantitative Profiling. Scientific
Reports 2023 13:1.

[45] van
der Laan T., Boom I., Maliepaard J., Dubbelman A. C., Harms A. C., Hankemeier T. (2020). Data-Independent
Acquisition for the Quantification and Identification of Metabolites
in Plasma. Metabolites.

[46] Lindemann V., Schmidt J., Cramer B., Humpf H. U. (2022). Detection of Mycotoxins
in Highly Matrix-Loaded House-Dust Samples by QTOF-HRMS, IM-QTOF-HRMS,
and TQMS: Advantages and Disadvantages. Anal.
Chem..

[47] Lodge S., Litton E., Gray N., Ryan M., Millet O., Fear M., Raby E., Currie A., Wood F., Holmes E., Wist J., Nicholson J. K. (2024). Stratification
of Sepsis Patients on Admission into the Intensive Care Unit According
to Differential Plasma Metabolic Phenotypes. J. Proteome Res..

[48] Lioupi A., Virgiliou C., Walter T. H., Smith K. M., Rainville P., Wilson I. D., Theodoridis G., Gika H. G. (2022). Application of a
Hybrid Zwitterionic Hydrophilic Interaction Liquid Chromatography
Column in Metabolic Profiling Studies. J. Chromatogr
A.

[49] Rimayi C., Chimuka L., Gravell A., Fones G. R., Mills G. A. (2019). Use of
the Chemcatcher® Passive Sampler and Time-of-Flight Mass Spectrometry
to Screen for Emerging Pollutants in Rivers in Gauteng Province of
South Africa. Environ. Monit Assess.

[50] Graça G., Cai Y., Lau C. H. E., Vorkas P. A., Lewis M. R., Want E. J., Herrington D., Ebbels T. M. D. (2022). Automated Annotation of Untargeted
All-Ion Fragmentation LC-MS Metabolomics Data with MetaboAnnotatoR. Anal. Chem..

[51] Johnson A. R., Carlson E. E. (2015). Collision-Induced
Dissociation Mass Spectrometry: A
Powerful Tool for Natural Product Structure Elucidation. Anal. Chem..

[52] Krupa S., Ruman T., Szuberla W., Nizioł J. (2025). Analysis of
the Spatial Distribution of Metabolites in Aloe Vera Leaves by Mass
Spectrometry Imaging and UHPLC-UHRMS. Scientific
Reports 2025 15:1.

[53] Szulc J., Grzyb T., Gutarowska B., Nizioł J., Krupa S., Ruman T. (2025). 3D Mass Spectrometry
Imaging as a
Novel Screening Method for Evaluating Biocontrol Agents. J. Agric. Food Chem..

[54] Szulc J., Grzyb T., Nizioł J., Krupa S., Szuberla W., Ruman T. (2025). Direct 3D Mass Spectrometry Imaging Analysis of Environmental Microorganisms. Molecules.

[55] Ruman T., Krupa Z., Nizioł J. (2024). Direct Three-Dimensional
Mass Spectrometry
Imaging with Laser Ablation Remote Atmospheric Pressure Photoionization/Chemical
Ionization. Anal. Chem..

[56] Arendowski A., Ossoliński K., Nizioł J., Ruman T. (2020). Screening of Urinary
Renal Cancer Metabolic Biomarkers with Gold Nanoparticles-Assisted
Laser Desorption/Ionization Mass Spectrometry. Anal. Sci..

[57] NIST Mass Spectral Libraries, 2023 Edition with Search Program, Data Version: NIST23, software version 3.0.

[58] Wishart D. S., Guo A. C., Oler E. (2022). HMDB 5.0: The Human
Metabolome Database for 2022. Nucleic Acids
Res..

[59] Nizioł J., Ossoliński K., Ossoliński T., Ossolińska A., Bonifay V., Sekuła J., Dobrowolski Z., Sunner J., Beech I., Ruman T. (2016). Surface-Transfer
Mass
Spectrometry Imaging of Renal Tissue on Gold Nanoparticle Enhanced
Target. Anal. Chem..

[60] Ossoliński K., Ruman T., Ossoliński T., Ossolińska A., Arendowski A., Kołodziej A., Płaza-Altamer A., Nizioł J. (2023). Monoisotopic Silver Nanoparticles-Based
Mass Spectrometry
Imaging of Human Bladder Cancer Tissue: Biomarker Discovery. Adv. Med. Sci..

[61] Zhang H., Lu K. H., Ebbini M., Huang P., Lu H., Li L. (2024). Mass Spectrometry Imaging
for Spatially Resolved Multi-Omics Molecular
Mapping. npj Imaging 2024 2:1.

[62] Kerwin J. L., Wiens A. M., Ericsson L. H. (1996). Identification
of Fatty Acids by
Electrospray Mass Spectrometry and Tandem Mass Spectrometry. Journal of Mass Spectrometry.

[63] Golley R. K., Hendrie G. A. (2014). Evaluation of the
Relative Concentration of Serum Fatty
Acids C14:0, C15:0 and C17:0 as Markers of Children’s Dairy
Fat Intake. Ann. Nutr Metab.

[64] Sun Q., Ma J., Campos H., Hu F. B. (2007). Plasma and Erythrocyte Biomarkers
of Dairy Fat Intake and Risk of Ischemic Heart Disease. Am. J. Clin. Nutr..

[65] Forouhi N. G., Koulman A., Sharp S. J., Imamura F., Kröger J., Schulze M. B., Crowe F. L., Huerta J. M., Guevara M., Beulens J. W. J., van
Woudenbergh G. J., Wang L., Summerhill K., Griffin J. L., Feskens E. J. M., Amiano P., Boeing H., Clavel-Chapelon F., Dartois L., Fagherazzi G., Franks P. W., Gonzalez C., Jakobsen M. U., Kaaks R., Key T. J., Khaw K. T., Kühn T., Mattiello A., Nilsson P. M., Overvad K., Pala V., Palli D., Quirós J. R., Rolandsson O., Roswall N., Sacerdote C., Sánchez M. J., Slimani N., Spijkerman A. M. W., Tjonneland A., Tormo M. J., Tumino R., van der A D. L., van der Schouw Y. T., Langenberg C., Riboli E., Wareham N. J. (2014). Differences
in the Prospective Association between Individual Plasma Phospholipid
Saturated Fatty Acids and Incident Type 2 Diabetes: The EPIC-InterAct
Case-Cohort Study. Lancet Diabetes Endocrinol.

[66] Warensjö E., Jansson J.-H., Berglund L., Boman K., Ahrén B., Weinehall L., Lindahl B., Hallmans G., Vessby B. (2004). Estimated
Intake of Milk Fat Is Negatively Associated with Cardiovascular Risk
Factors and Does Not Increase the Risk of a First Acute Myocardial
Infarction. A Prospective Case-Control Study. Br. J. Nutr..

[67] Liss M. A., Al-Bayati O., Gelfond J., Goros M., Ullevig S., DiGiovanni J., Hamilton-Reeves J., O’Keefe D., Bacich D., Weaver B., Leach R., Thompson I. M. (2019). Higher
Baseline Dietary Fat and Fatty Acid Intake Is Associated with Increased
Risk of Incident Prostate Cancer in the SABOR Study. Prostate Cancer and Prostatic Diseases 2018 22:2.

[68] Sczaniecka A. K., Brasky T. M., Lampe J. W., Patterson R. E., White E. (2012). Dietary Intake of Specific Fatty
Acids and Breast Cancer Risk Among
Postmenopausal Women in the VITAL Cohort. Nutr
Cancer.

[69] Wu Q., Shi D., Dong T., Zhang Z., Ou Q., Fang Y., Zhang C. (2023). Serum Saturated Fatty Acids Including
Very Long-Chain Saturated Fatty
Acids and Colorectal Cancer Risk among Chinese Population. Nutrients.

[70] Kim H. Y., Moon J. Y., Cho S. K. (2023). Heptadecanoic Acid, an Odd-Chain
Fatty Acid, Induces Apoptosis and Enhances Gemcitabine Chemosensitivity
in Pancreatic Cancer Cells. https://home.liebertpub.com/jmf.

[71] Fajardo V. A., McMeekin L., Leblanc P. J. (2011). Influence of Phospholipid Species
on Membrane Fluidity: A Meta-Analysis for a Novel Phospholipid Fluidity
Index. J. Membr. Biol..

[72] Li S., Yuan H., Li L., Li Q., Lin P., Li K. (2025). Oxidative Stress and Reprogramming
of Lipid Metabolism in Cancers. Antioxidants.

[73] Brandi J., Dando I., Pozza E. D., Biondani G., Jenkins R., Elliott V., Park K., Fanelli G., Zolla L., Costello E., Scarpa A., Cecconi D., Palmieri M. (2017). Proteomic
Analysis of Pancreatic Cancer Stem Cells: Functional Role of Fatty
Acid Synthesis and Mevalonate Pathways. J. Proteomics.

[74] Zemanová M., Vecka M., Petruželka L., Staňková B., Žák A., Zeman M. (2016). Plasma Phosphatidylcholines Fatty
Acids in Men with Squamous Cell Esophageal Cancer: Chemoradiotherapy
Improves Abnormal Profile. Med. Sci. Monit.

[75] dos
Reis Riccardi D. M., das Neves R. X., de Matos-Neto E. M., Camargo R. G., Lima J. D. C. C., Radloff K., Alves M. J., Costa R. G. F., Tokeshi F., Otoch J. P., Maximiano L. F., de Alcantara P. S. M., Colquhoun A., Laviano A., Seelaender M. (2020). Plasma Lipid
Profile and Systemic Inflammation in Patients With Cancer Cachexia. Front. Nutr..

[76] Mei J., Qian M., Hou Y., Liang M., Chen Y., Wang C., Zhang J. (2024). Association
of Saturated Fatty Acids
with Cancer Risk: A Systematic Review and Meta-Analysis. Lipids Health Dis.

[77] Feng C., Pan L., Tang S., He L., Wang X., Tao Y., Xie Y., Lai Z., Tang Z., Wang Q., Li T. (2021). Integrative
Transcriptomic, Lipidomic, and Metabolomic Analysis Reveals Potential
Biomarkers of Basal and Luminal Muscle Invasive Bladder Cancer Subtypes. Front Genet.

[78] Pereira F., Domingues M. R., Vitorino R., Guerra I. M. S., Santos L. L., Ferreira J. A., Ferreira R. (2024). Unmasking the Metabolite Signature
of Bladder Cancer: A Systematic Review. Int.
J. Mol. Sci..

[79] Jin Q., Qi D., Zhang M., Qu H., Dong Y., Sun M., Quan C. (2024). CLDN6 Inhibits Breast Cancer Growth and Metastasis through SREBP1-Mediated
RAS Palmitoylation. Cell Mol. Biol. Lett..

[80] Li J., Cheng B., Xie H., Zhan C., Li S., Bai P. (2022). Bladder Cancer Biomarker
Screening Based on Non-Targeted Urine Metabolomics. Int. Urol Nephrol.

[81] D’Aniello C., Patriarca E. J., Phang J. M., Minchiotti G. (2020). Proline Metabolism
in Tumor Growth and Metastatic Progression. Front Oncol.

[82] Elia I., Broekaert D., Christen S., Boon R., Radaelli E., Orth M. F., Verfaillie C., Grünewald T. G.
P., Fendt S. M. (2017). Proline
Metabolism Supports Metastasis Formation and
Could Be Inhibited to Selectively Target Metastasizing Cancer Cells. Nature Communications 2017 8:1.

[83] Ossoliński K., Ruman T., Copié V., Tripet B. P., Nogueira L. B., Nogueira K. O. P. C., Kołodziej A., Płaza-Altamer A., Ossolińska A., Ossoliński T., Nizioł J. (2022). Metabolomic
and Elemental Profiling of Blood Serum in Bladder Cancer. J. Pharm. Anal.

[84] Wang R., Kang H., Zhang X., Nie Q., Wang H., Wang C., Zhou S. (2022). Urinary Metabolomics
for Discovering
Metabolic Biomarkers of Bladder Cancer by UPLC-MS. BMC Cancer.

[85] Suda A., Umaru B. A., Yamamoto Y., Shima H., Saiki Y., Pan Y., Jin L., Sun J., Low Y. L. C., Suzuki C., Abe T., Igarashi K., Furukawa T., Owada Y., Kagawa Y. (2024). Polyunsaturated
Fatty Acids-Induced Ferroptosis Suppresses Pancreatic Cancer Growth. Scientific Reports 2024 14:1.

[86] Yang X., Liu Y., Wang Z., Jin Y., Gu W. (2024). Ferroptosis as a New
Tool for Tumor Suppression through Lipid Peroxidation. Communications Biology 2024 7:1.

[87] Borgonovi S. M., Iametti S., Di Nunzio M. (2023). Docosahexaenoic
Acid as Master Regulator of Cellular Antioxidant Defenses: A Systematic
Review. Antioxidants.

[88] Tatsumi Y., Kato A., Niimi N., Yako H., Himeno T., Kondo M., Tsunekawa S., Kato Y., Kamiya H., Nakamura J., Higai K., Sango K., Kato K. (2022). Docosahexaenoic
Acid Suppresses Oxidative Stress-Induced Autophagy and Cell Death
via the AMPK-Dependent Signaling Pathway in Immortalized Fischer Rat
Schwann Cells 1. Int. J. Mol. Sci..

[89] Tamura K., Horikawa M., Sato S., Miyake H., Setou M. (2019). Discovery
of Lipid Biomarkers Correlated with Disease Progression in Clear Cell
Renal Cell Carcinoma Using Desorption Electrospray Ionization Imaging
Mass Spectrometry. Oncotarget.

[90] D’Eliseo D., Manzi L., Merendino N., Velotti F. (2012). Docosahexaenoic Acid
Inhibits Invasion of Human RT112 Urinary Bladder and PT45 Pancreatic
Carcinoma Cells via Down-Modulation of Granzyme B Expression. J. Nutr Biochem.

[91] Ding Y., Yang J., Ma Y., Yao T., Chen X., Ge S., Wang L., Fan X. (2019). MYCN and PRC1
Cooperatively Repress
Docosahexaenoic Acid Synthesis in Neuroblastoma via ELOVL2. Journal of Experimental and Clinical Cancer Research.

[92] Li Y., Xu H., Lin T., Zhang J., Ai J., Zhang S., Le W., Tan P., Zhang P., Wei Q., Zheng X., Yang L. (2024). Preoperative
Low Plasma Creatine Kinase Levels Predict Worse Survival
Outcomes in Bladder Cancer after Radical Cystectomy. Int. Urol Nephrol.

[93] Maguire O. A., Ackerman S. E., Szwed S. K., Maganti A. V., Marchildon F., Huang X., Kramer D. J., Rosas-Villegas A., Gelfer R. G., Turner L. E., Ceballos V., Hejazi A., Samborska B., Rahbani J. F., Dykstra C. B., Annis M. G., Luo J. D., Carroll T. S., Jiang C. S., Dannenberg A. J., Siegel P. M., Tersey S. A., Mirmira R. G., Kazak L., Cohen P. (2021). Creatine-Mediated Crosstalk between
Adipocytes and Cancer Cells Regulates
Obesity-Driven Breast Cancer. Cell Metab.

[94] Maqdasy S., Lecoutre S., Renzi G., Frendo-Cumbo S., Rizo-Roca D., Moritz T., Juvany M., Hodek O., Gao H., Couchet M., Witting M., Kerr A., Bergo M. O., Choudhury R. P., Aouadi M., Zierath J. R., Krook A., Mejhert N., Rydén M. (2022). Impaired Phosphocreatine Metabolism
in White Adipocytes Promotes Inflammation. Nature
Metabolism 2022 4:2.

[95] Nizioł J., Copié V., Tripet B. P., Nogueira L. B., Nogueira K. O. P. C., Ossoliński K., Arendowski A., Ruman T. (2021). Metabolomic and Elemental Profiling of Human Tissue in Kidney Cancer. Metabolomics.

[96] Sun C., Wang F., Zhang Y., Yu J., Wang X. (2020). Mass Spectrometry
Imaging-Based Metabolomics to Visualize the Spatially Resolved Reprogramming
of Carnitine Metabolism in Breast Cancer. Theranostics.

[97] Vallianatou T., Shariatgorji R., Nilsson A., Karlgren M., Hulme H., Fridjonsdottir E., Svenningsson P., Andrén P. E. (2021). Integration
of Mass Spectrometry Imaging and Machine Learning Visualizes Region-Specific
Age-Induced and Drug-Target Metabolic Perturbations in the Brain. ACS Chem. Neurosci..

[98] Liu Z., Fan M., Hou J., Pan S., Xu Y., Zhang H., Liu C., Hao X., Li X., Wang H. (2023). Serine Hydroxymethyltransferase
2 Knockdown Induces Apoptosis in CcRCC by Causing Lysosomal Membrane
Permeabilization via Metabolic Reprogramming. Cell Death & Disease 2023 14:2.

[99] Putluri N., Shojaie A., Vasu V. T., Vareed S. K., Nalluri S., Putluri V., Thangjam G. S., Panzitt K., Tallman C. T., Butler C., Sana T. R., Fischer S. M., Sica G., Brat D. J., Shi H., Palapattu G. S., Lotan Y., Weizer A. Z., Terris M. K., Shariat S. F., Michailidis G., Sreekumar A. (2011). Metabolomic
Profiling Reveals Potential
Markers and Bioprocesses Altered in Bladder Cancer Progression. Cancer Res..

[100] Dettmer K., Vogl F. C., Ritter A. P., Zhu W., Nürnberger N., Kreutz M., Oefner P. J., Gronwald W., Gottfried E. (2013). Distinct Metabolic Differences between
Various Human
Cancer and Primary Cells. Electrophoresis.

[101] Zhang J., Ruan K., Chu Z., Wang X., Gu Y., Jin H., Zhang X., Liu Q., Yang J. (2025). Reprogramming
of Fatty Acid Metabolism: A Hidden Force Regulating the Occurrence
and Progression of Cholangiocarcinoma. Cell
Death Discovery.

[102] Jin R., Dai Y., Wang Z., Hu Q., Zhang C., Gao H., Yan Q. (2025). Unraveling Ferroptosis:
A New Frontier in Combating
Renal Fibrosis and CKD Progression. Biology.

[103] Ortiz-Placin C., Castillejo-Rufo A., Estaras M., Gonzalez A. (2023). Membrane Lipid
Derivatives: Roles of Arachidonic Acid and Its Metabolites in Pancreatic
Physiology and Pathophysiology. Molecules.

[104] Tredicine M., Mucci M., Recchiuti A., Mattoscio D. (2025). Immunoregulatory Mechanisms of the Arachidonic Acid
Pathway in Cancer. FEBS Lett..

[105] Kim J. W., Lee J. Y., Oh M., Lee E. W. (2023). An Integrated
View of Lipid Metabolism in Ferroptosis Revisited via Lipidomic Analysis. Experimental & Molecular Medicine 2023 55:8.

[106] Arendowski A., Nizioł J., Ossoliński K., Ossolińska A., Ossoliński T., Dobrowolski Z., Ruman T. (2018). Laser Desorption/Ionization
MS Imaging of Cancer Kidney Tissue on
Silver Nanoparticle-Enhanced Target. Bioanalysis.

