# Supplementary material for: Broadband Collision-Induced Dissociation Mass Spectrometry Imaging
Source: J Am Soc Mass Spectrom. 2025 Jun 19;36(7):1443–55. doi: 10.1021/jasms.5c00045 (PMC12232394; doi:10.1021/jasms.5c00045)
Supplement: Supplementary file 1 [file js5c00045_si_001.pdf]

Supporting Information

Broadband collision-induced dissociation mass spectrometry imaging

Sumi Krupa[1], Wiktoria Szuberla[2], Joanna Nizioł[3*], Anna Ossolińska[4], Krzysztof Ossoliński[4] and Tomasz Ruman[2]

[1]*Doctoral School at the Rzeszów University of Technology, 8 Powstańców Warszawy Ave., 35-959 Rzeszów, Poland*

[2]*Department of Inorganic and Analytical Chemistry, Faculty of Chemistry, Rzeszów University of Technology, 6 Powstańców Warszawy Ave., Rzeszów, 35-959, Poland*

[3]*Department of Polymers and Biopolymers, Faculty of Chemistry, Rzeszów University of Technology, 6 Powstańców Warszawy Ave., Rzeszów, 35-959, Poland*

[4]*Department of Urology, John Paul II Hospital, Grunwaldzka 4 St., Kolbuszowa, 36-100, Poland*

*Corresponding author: Joanna Nizioł, e-mail: jniziol@prz.edu.pl, tel: (+48 17) 8651550

Table of Contents

FS1. The results of positive mode LARAPPI/CI-MSI analysis. Ion images of precursor and fragment ions of each compound are shown above the ESI MS/MS spectra.

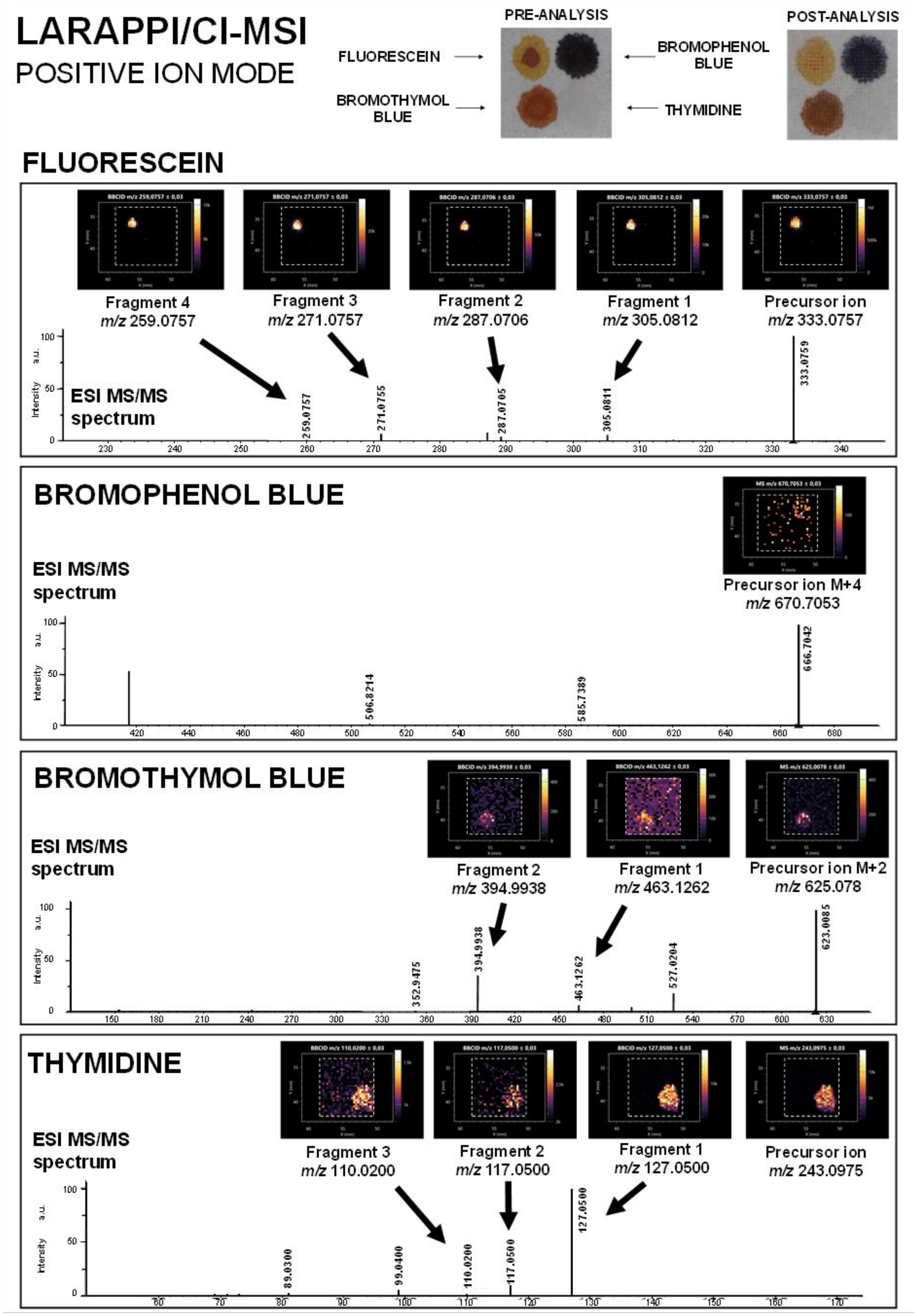

Fig. S1. The results of positive mode LARAPPI/CI-MSI analysis. Ion images of precursor and fragment ions of each compound are shown above the ESI MS/MS spectra.
